# The HEAT Repeat Protein MROH1 Deficiency Leads to Reduced Circulating Thyroid Hormone Levels in Mice

**DOI:** 10.1002/edm2.70348

**Published:** 2026-09-18

**Authors:** Nami Ohuchi, Yoshinori Osaki, Yoshimi Nakagawa, Takafumi Miyamoto, Masaya Araki, Yuhei Mizunoe, Takaaki Matsuda, Yuki Murayama, Yoko Sugano, Hitoshi Iwasaki, Takashi Matsuzaka, Motohiro Sekiya, Hitoshi Shimano

**Affiliations:** ^1^ Department of Endocrinology and Metabolism Institute of Medicine, University of Tsukuba Tsukuba Ibaraki Japan; ^2^ Division of Complex Biosystem Research, Department of Research and Development, Institute of Natural Medicine University of Toyama Toyama Japan; ^3^ Laboratory of Molecular Pathology & Metabolic Disease, Faculty of Pharmaceutical Sciences Tokyo University of Science Noda Chiba Japan; ^4^ Department of Endocrinology and Metabolism University of Tsukuba Hospital Tsukuba Ibaraki Japan

**Keywords:** hypothyroidism, knockout, mice, thyroid gland

## Abstract

**Introduction:**

Systemic metabolism critically depends on thyroid hormones, whose release relies heavily on proper lysosomal function for thyroglobulin degradation. MROH1, a highly conserved mediator of lysosomal membrane fission, is predominantly expressed in the human thyroid gland; however, its physiological role within endocrine systems remains unexplored. This study investigated the impact of MROH1 deficiency on thyroid function using a novel global *Mroh1* knockout (KO) mouse model.

**Methods:**

Metabolic phenotypes, including body weight and serum biochemical parameters (FT3, FT4, TSH, and cholesterol), were evaluated in male *Mroh1*‐KO mice and their controls. Thyroid architecture and gene expression profiles were investigated via histological analysis and quantitative reverse transcription‐PCR (qRT‐PCR). Lysosomal function was assessed using immunoblotting and cathepsin L proteolytic activity assays in primary thyroid cells and whole‐tissue lysates.

**Results:**

*Mroh1*‐KO mice showed reduced circulating thyroid hormone levels characterized by significantly reduced serum FT4 and FT3 levels, a gradual increase in body weight, and hypercholesterolemia. Histological analysis of aged *Mroh1*‐KO mice revealed an altered thyroid architecture, featuring smaller follicle sizes and the replacement of follicles with adipocyte‐like structures. Although lysosomal biogenesis and cathepsin L protease activities remained preserved, the expression of key thyroid differentiation regulators (*Nkx2‐1*, *Foxe1*) and their downstream targets (*Tg*, *Mct8*) were significantly downregulated.

**Conclusions:**

Global *Mroh1* deficiency is associated with reduced circulating thyroid hormone levels, thyroid architectural remodelling, and downregulated thyroid‐specific gene expression. These findings demonstrate that MROH1 is important for maintaining thyroid gland hormone homeostasis and structural integrity.

## Graphical abstract

Global *Mroh1* deficiency in male mice is associated with reduced circulating thyroid hormone level, late‐onset thyroid follicular remodelling, and lower whole‐thyroid abundance of Nkx2‐1, Foxe1, and Tg transcripts. These findings support a role for MROH1 in maintaining thyroid homeostasis and structural integrity.

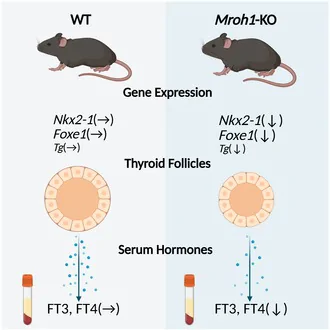

## 1 Introduction

Efficient systemic metabolic control relies on thyroid hormones (predominantly thyroxine, T4, and triiodothyronine, T3). The biosynthesis of these hormones requires the iodination of thyroglobulin (Tg) within follicular lumens, followed by endocytosis of Tg into follicular cells. Importantly, subsequent hormone release is contingent upon lysosomal proteolytic degradation [1]. Consequently, the proper regulation of the lysosomal system, including its membrane trafficking and structural dynamics, is paramount for efficient hormone liberation.

To sustain their functional integrity, lysosomes undergo continuous, highly regulated cycles of fusion and fission [2]. Recent studies have identified the conserved HEAT‐repeat protein HPO‐27 in *Caenorhabditis elegans* and its human homologue, MROH1 (HEATR7A), as critical mediators of lysosomal membrane fission [2]. In *C. elegans* , the loss of HPO‐27 results in the accumulation of aberrant tubular lysosomal networks in multiple tissues, including the hypodermis, intestine, and body wall muscle cells, rather than the typical vesicular structures [2].

MROH1 is a novel regulator of lysosomal scission through its interaction with the WASH‐actin machinery [3]. However, its physiological role in endocrine systems—where lysosomal function is particularly crucial—remains unexplored. In this study, we investigated the impact of MROH1 deficiency on thyroid function.

To date, no studies have implicated MROH1 in thyroid biology. In fact, the association between lysosomal membrane dynamics and endocrine function remains largely uncharacterized. Interestingly, transcriptomic profiling across large human gene expression databases, including GTEx and the Human Protein Atlas, classifies MROH1 expression as markedly “tissue‐enhanced” in the thyroid gland relative to other organs [4, 5]. This finding—preferential expression of a core lysosomal fission factor in an endocrine gland—strongly suggests a potential role of MROH1 in thyroid pathophysiology. Therefore, we generated a global *MROH1* knockout mouse model to investigate its physiological role.

Here, we demonstrate that *MROH1* deficiency leads to a mild hypothyroidism phenotype, characterized by significantly reduced circulating FT4 and FT3 levels and hypercholesterolemia. Importantly, although baseline lysosomal enzymatic activity and protein expression levels appeared unchanged, histological analysis revealed structural alterations within the thyroid tissue. Together, these findings establish MROH1 as an important genetic factor for maintaining thyroid hormone homeostasis and tissue integrity.

## 2 Materials and Methods

### 2.1 Animals

All animal experiments were conducted in accordance with the Fundamental Guidelines for Proper Conduct of Animal Experiments and Related Activities in Academic Research Institutions (Ministry of Education, Culture, Sports, Science and Technology, Japan). Protocols were approved by the Animal Care and Use Committee (approval number: 24‐088) and the Gene Modification Experiment Safety Committee (approval number: 210255) of the University of Tsukuba.

Sperm carrying the *Mroh1* ^*tm1a(KOMP)Wtsi*^ allele were obtained from the European Mouse Mutant Archive (EMMA) [6, 7, 8, 9]. To generate *Mroh1*‐floxed mice, in vitro fertilization was performed using C57BL/6J oocytes, followed by electroporation of *Flpe* mRNA into fertilized eggs to excise the FRT‐flanked gene trap cassette, as previously described [10]. Global *Mroh1*‐deficient mice (*Mroh1* ^−/−^, hereafter *Mroh1*‐KO mice) were generated by crossing *Mroh1*‐floxed mice with *Ayu1‐Cre* transgenic mice, resulting in the ubiquitous excision of exons 5–7. Cre‐negative mice homozygous for the floxed allele (*Mroh1* ^fl/fl^) served as wild‐type controls (Ctrl). Male mice were randomly allocated to experimental groups and used for all animal experiments. Primer sequences for detecting the *Mroh1*‐flox allele are listed in Table S1.

### 2.2 Physiological Analysis

Body weight was measured every 4 weeks from 4 to 28 weeks of age, and body length (naso‐anal length) was determined at 24 weeks of age. To analyse food intake, 6–7‐month‐old mice were housed individually. After a one‐week acclimatization period, cumulative food consumption was measured over the subsequent 7 days.

### 2.3 RNA Extraction and Gene Expression Analysis

Total RNA was isolated from thyroid and other designated tissues using Sepasol‐RNA I Super G (Nacalai Tesque Inc.) according to the manufacturer's instructions. RNA was reverse‐transcribed using the PrimeScript RT Master Mix (Takara Bio Inc.). Quantitative reverse‐transcription (qRT)‐PCR was performed using SYBR Green qPCR Master Mix (Thermo Fisher Scientific) on a LightCycler 96 System (Roche Diagnostics). Relative target gene expression was calculated using the ΔΔCt method, normalized to *Gapdh* expression as an internal reference. Primer sequences used in this study are listed in Table S2.

### 2.4 Serum Biochemical Analysis

Blood samples were collected, and serum was isolated via centrifugation (1000 × g, 10 min, 4°C). Serum concentrations of total cholesterol, triglycerides, aspartate aminotransferase (AST), alanine aminotransferase (ALT), creatinine, creatine kinase (CK), and glucose were measured using commercially available assay kits (FUJIFILM Wako Pure Chemical Corporation, Osaka, Japan) according to the manufacturers' instructions (catalogue numbers: #439‐17,501, #432‐40,201, #431‐30,901, #636‐51,011, #464‐43,601, #439‐90,901, respectively).

Serum free T3 (FT3) levels were measured via a chemiluminescent enzyme immunoassay (CLEIA) using the Accuraseed Free T3 kit (FUJIFILM Wako Pure Chemical Corporation, Osaka, Japan). Serum free T4 (FT4) and TSH levels were quantified using specific ELISA kits (CSB‐E05080m, CUSABIO TECHNOLOGY LLC; and CEA463Mu, Cloud‐Clone Corp., respectively) per the manufacturers' instructions.

### 2.5 Histological Analysis

Following euthanasia, the thyroid gland, brain, lung, liver, and tibialis anterior muscle were harvested, fixed overnight in 10% neutral buffered formalin, and paraffin‐embedded using standard procedures. Tissue sections (5 μm thickness) were stained with haematoxylin and eosin (H&E). Brightfield imaging was performed using either a Nikon ECLIPSE Ts2R microscope equipped with a Nikon DS‐Ri2 digital camera or a Keyence BZ‐X710 microscope.

### 2.6 Quantification of Thyroid Follicle Area and Size Distribution

Thyroid follicle area and size distribution were quantified as previously described [11] using ImageJ/Fiji software (version 1.54p; National Institute of Health). First, non‐thyroidal tissues, such as the trachea and muscles, were manually excluded from H&E images. Thyroid follicles were then segmented using the Trainable Weka Segmentation plugin [12] trained to distinguish follicles from the interstitium and other tissues. Probability maps were converted into binary masks, followed by noise reduction (< 24 μm^2^) and hole filling.

To evaluate follicle morphology, the cross‐sectional area of each individual follicle was extracted using the “Analyse Particles” function, and the total area of the segmented follicles (Area_Follicle Total_) was determined as the sum of these individual measurements.

The total area of the thyroid gland (Area_Thyroid Total_) was measured by manually outlining the thyroid lobes. Finally, the follicle area ratio (%) was calculated as follows:

Follicle Area Ratio (%) = (Area_Follicle Total_/Area_Thyroid Total_) × 100.

### 2.7 Immunoblotting

Tissue lysates were prepared in Mammalian Protein Extraction Buffer (Cytiva, formerly GE Healthcare Bio‐Sciences Corp., Marlborough, MA, USA) as described previously [13, 14]. Homogenized tissues were centrifuged at 15,000 × g for 10 min, and supernatants were collected. Total protein concentrations from the thyroid tissues were determined using a Protein Assay BCA Kit (06385‐00; Nacalai, Kyoto, Japan). Equal protein aliquots were resolved using SDS‐PAGE and transferred to polyvinylidene fluoride membranes. The membranes were probed with the appropriate primary antibodies (Table S3) overnight at 4°C, followed by a 1‐h incubation with the appropriate secondary antibodies at room temperature. Protein bands were visualized using Clarity Western ECL Substrate (#1705061; Bio‐Rad, Hercules, CA, USA) and imaged on a ChemiDoc XRS Plus system (170‐8265J1PC; Bio‐Rad).

### 2.8 Cell Preparation and Functional Analysis of Cathepsin

Primary thyroid cells were isolated from 9‐ to 13‐week‐old mice via collagenase digestion. Passage 2 (P2) cells were seeded into 96‐well plates at a density of 1 × 10^4^ cells per well and assayed after 24 h.

Live‐cell cathepsin L activity was measured using the Magic Red Cathepsin L Assay Kit (ab270774, Abcam), with signals normalized to Hoechst 33342 nuclear staining (H3570, Thermo Fisher Scientific). For whole‐tissue analyses, thyroid tissue sections (approximately 10 mg per sample) from 6‐month‐old mice were processed, and cathepsin L activity was determined using the Cathepsin L Activity Assay Kit (CBA023, Merck) in accordance with the manufacturer's instructions. All fluorescence intensity measurements were performed using a Synergy HTX Multi‐Mode Reader (BioTek Instruments).

### 2.9 Statistical Analyses

Data are expressed as the mean ± standard error of the mean. Statistical comparisons between two groups were performed using an unpaired Student's *t*‐test. Differences in the size distribution of individual follicles were evaluated using the Kolmogorov–Smirnov test. All analyses were performed in GraphPad Prism 10 (GraphPad Software Inc., Boston, MA, USA). *p*‐values < 0.05 (two‐tailed) were considered statistically significant.

## 3 Results

### 3.1 Generation and Characterization of *Mroh1*‐Deficient Mice

**FIGURE 1:**
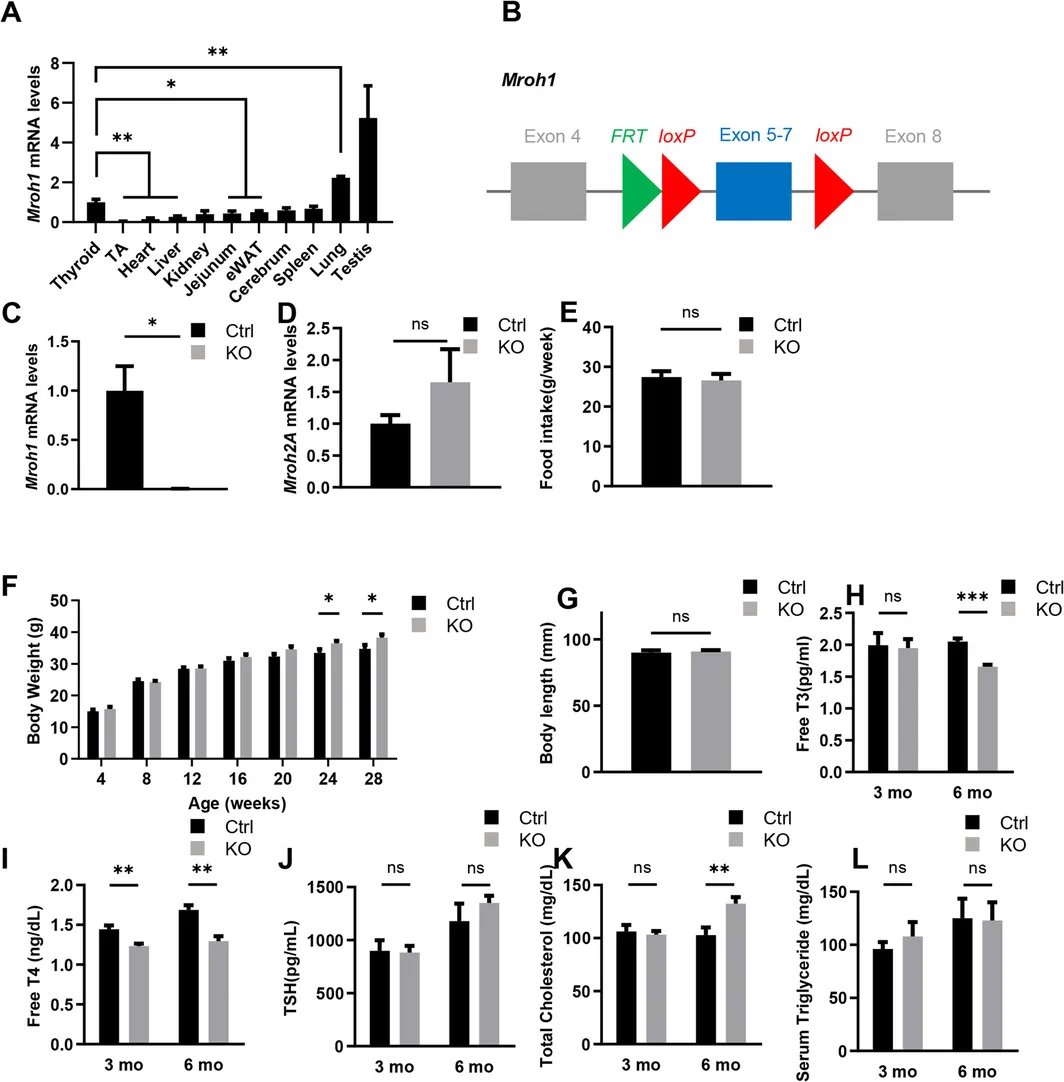
Generation and phenotypic characterization of *Mroh1* knockout mice. (A) Tissue distribution of *Mroh1* mRNA. *Mroh1* mRNA expression levels in various tissues of control (Ctrl) mice were measured using quantitative RT‐PCR (*n* = 3). Statistical significance for each tissue was determined by comparison to the thyroid. (B) Schematic representation of the *Mroh1* gene‐targeting strategy. (C) Confirmation of *Mroh1* mRNA deletion. *Mroh1* mRNA expression levels in the thyroids of Ctrl and *Mroh1* knockout (KO) mice (*n* = 3 per group). (D) *Mroh2a* mRNA expression levels. Relative *Mroh2a* expression in the thyroids of Ctrl (*n* = 7) and KO mice (*n* = 6). (E) Weekly food intake monitored in Ctrl and KO mice (*n* = 3 per group). (F) Growth curve. Body weights of Ctrl and KO mice from 4 to 28 weeks of age (*n* = 6–19 per group). (G) Body length. Naso‐anal length of Ctrl and KO mice measured at 24 weeks of age (*n* = 4 per group). Serum biochemical and hormonal profiles. Serum levels of (H) FT3, (I) FT4, (J) TSH, (K) total cholesterol, and (L) triglycerides (TG) in Ctrl and KO mice at 3 and 6 months of age. (*n* = 3–7 per group). Data are presented as the mean ± SEM. Statistical significance was determined using Student's *t*‐test. In (A), each tissue was compared with the thyroid. In other panels, Ctrl and KO groups were compared. *p < 0.05, **p < 0.01, ***p < 0.001, ns, not significant. Ctrl, control; eWAT, epididymal white adipose tissue; KO, knockout; mo, month(s); RT‐PCR, reverse transcription‐PCR; TA, tibialis anterior muscle.

Given the GTEx portal database indicating predominant *MROH1* expression in the human thyroid gland [4], we investigated its physiological role in the thyroid tissue. First, we examined the tissue distribution of *Mroh1* mRNA in Ctrl mice using qRT‐PCR. *Mroh1* expression was highest in the testis, followed by the lung and the thyroid gland (Figure 1A). This distribution differs slightly from that in humans, where MROH1 expression is restricted to the thyroid [4].

To elucidate the physiological function of MROH1, we analysed global *Mroh1*‐deficient mice (KO mice). The gene‐targeting strategy is schematically illustrated in Figure 1B. Quantitative RT‐PCR analysis confirmed the complete absence of *Mroh1* transcripts in the thyroids of KO mice (Figure 1C).

*Mroh2a* is an important paralog of *Mroh1* with high sequence homology. To investigate potential functional compensation by *Mroh2a*, a highly homologous paralog of *Mroh1*, we examined its expression levels in the thyroid. Although *Mroh2a* mRNA levels exhibited an increasing trend in the KO group, the difference compared to controls was not significant (Figure 1D).

Metabolic phenotyping revealed no significant differences in food intake between genotypes or body length between genotypes (Figure 1E,G). However, KO mice exhibited a gradual increase in body weight, reaching statistical significance by 24 weeks of age compared to Ctrl (Figure 1F).

Next, we assessed thyroid function by measuring serum hormone levels. Serum FT3 and FT4 levels were significantly reduced in KO mice compared to Ctrl, with FT4 being lower at both 3 and 6 months and FT3 being lower at 6 months of age (Figure 1H,I). Serum TSH levels showed a non‐significant increasing trend in KO mice compared to Ctrl (Figure 1J).

Because hypothyroidism is clinically associated with dyslipidemia, particularly hypercholesterolemia, we analysed serum lipid profiles. Consistent with a mild hypothyroid phenotype, total serum cholesterol levels were comparable to Ctrl at 3 months but were significantly elevated by 6 months of age in KO mice (Figure 1K). In contrast, serum triglyceride levels remained unchanged between Ctrl and KO mice (Figure 1L). Markers of liver and kidney function (AST, ALT, and creatinine), blood glucose, and CK levels were also comparable between Ctrl and KO mice (Figure S1). Furthermore, histological examination revealed no overt abnormalities in other major tissues, including the lung, brain, liver, and skeletal muscle (Figure S2).

### 3.2 Histological and Molecular Analysis of the *Mroh1*‐Deficient Thyroid

**FIGURE 2:**
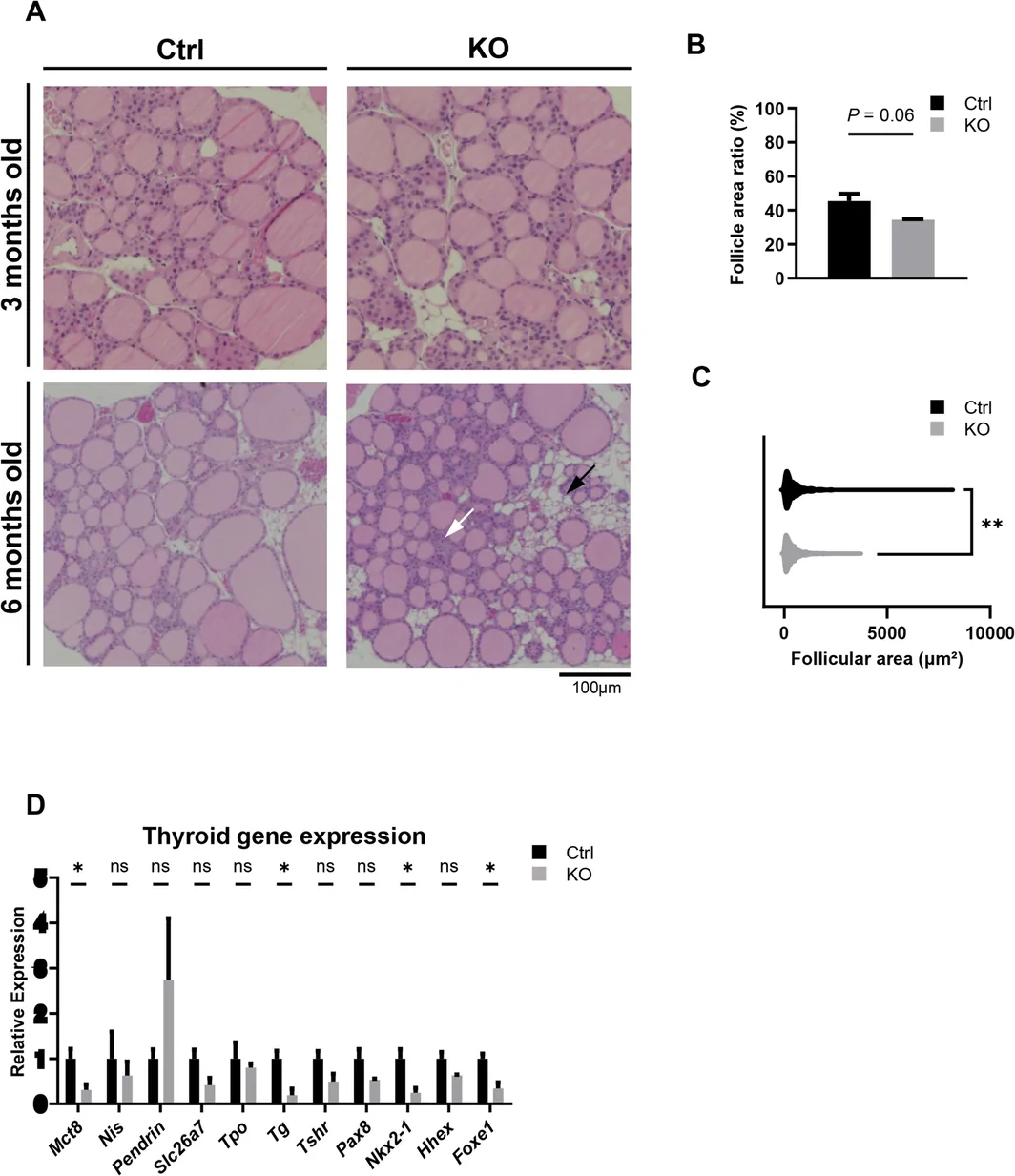
Histological and molecular analysis of the *Mroh1*‐deficient thyroid. (A) Representative images of H&E‐stained thyroid sections. Thyroid tissues were harvested from Ctrl and KO mice at 3 and 6 months of age. At 6 months, KO mice exhibited altered tissue architecture characterized by reduced follicle area and the replacement of follicles with adipocyte‐like structures (black arrows) and interstitial cells (white arrowheads). Scale bar = 100 μm. (B) Thyroid follicle area ratio. The percentage of follicle area relative to the total thyroid area in Ctrl and KO mice at 6 months of age (*n* = 3 per group). The *p*‐value was determined by Student's *t*‐test. (C) Thyroid follicle size distribution. Violin plots showing the size distribution of individual thyroid follicles in Ctrl and KO mice (*n* = 837 follicles from 3 Ctrl mice and *n* = 895 follicles from 3 KO mice). Statistical significance of the distribution shift was evaluated using the Kolmogorov‐Smirnov test. (D) mRNA expression levels of thyroid‐related genes. Relative mRNA expression levels of *Slc16a2* (MCT8), *Slc5a5* (NIS), *Slc26a4* (Pendrin), *Slc26a7*, *Tpo*, *Tg*, *Tshr*, *Pax8*, *Nkx2‐1*, *Hhex*, and *Foxe1* in the thyroid of Ctrl and KO mice at 6 months of age, measured using quantitative RT‐PCR (*n* = 3 per group). Data are presented as the mean ± SEM. Statistical significance was determined using Student's *t*‐test. *p < 0.05, **p < 0.01, ns, not significant. Ctrl, control; H&E, haematoxylin and eosin; KO, knockout.

To investigate the structural basis of the mild hypothyroid phenotype (Figure 1), we performed histological analysis of the thyroid gland using H&E staining. At 3 months of age, thyroid morphology in KO mice was comparable to that of Ctrl mice. By 6 months of age, however, KO mice exhibited altered tissue architecture characterized by a reduced follicular area and the replacement of follicles with interstitial cells and adipocyte‐like structures (Figure 2A). Quantification of the follicle area ratio (follicle area normalized to total thyroid area) supported these histological observations, showing a trend towards a decreased follicle area ratio at 6 months of age compared to Ctrl mice; however, the difference did not reach statistical significance (Figure 2B). To address whether overall organ‐level averaging might mask subtle cellular heterogeneity, we further evaluated the size distribution of individual follicles. Visualization using a violin plot combined with the Kolmogorov–Smirnov test revealed a statistically significant shift towards smaller follicle sizes in KO mice compared to Ctrl mice (*p* = 0.0095; *n* = 837 follicles from 3 Ctrl mice and *n* = 895 follicles from 3 KO mice, Figure 2C), demonstrating that loss of *Mroh1* results in a structural shift towards smaller thyroid follicles.

To investigate the molecular basis of the observed thyroid dysfunction, we analysed gene expression levels in isolated thyroid tissues. Quantitative RT‐PCR analysis revealed that the expression of *Nkx2‐1* and *Foxe1*, key regulators of thyroid differentiation, was significantly reduced in KO mice (Figure 2D), with *Pax8* and *Hhex* exhibiting a similar downward trend. Concurrently, the expression of *Tg*, the major precursor of thyroid hormone synthesis, was significantly decreased in KO mice (Figure 2D). Furthermore, the expression of *Slc16a2* (*Mct8*), a thyroid hormone transporter, was significantly downregulated, while other genes involved in hormone synthesis and transport, such as *Tshr*, *Slc5a5* (*Nis*), and *Slc26a7*, exhibited decreasing trends (Figure 2D).

These results suggest that MROH1 deficiency may influence the long‐term maintenance of thyroid follicular cells, potentially involving alterations in their proliferation or differentiation at later ages. The later structural changes and reduced whole‐thyroid abundance of transcripts related to thyroid hormone synthesis could contribute to the reduced circulating thyroid hormone levels in these mice.

### 3.3 Lysosomal Protein Abundance and Cathepsin L Activity Are Preserved in *Mroh1*‐Deficient Thyroid

Thyroid hormone release depends on the lysosomal proteolytic degradation of Tg, a process known to be mediated by lysosomal proteases, particularly cathepsins (e.g., cathepsin B, L, and K) [15, 16]. Furthermore, a recent study reported that MROH1 plays a critical role in maintaining lysosomal integrity. In *C. elegans* and mammalian COS‐7 cells, depletion of *Mroh1* (or its homologue) induces lysosomal structural defects and impairs the proteolytic activity of cathepsin L [2].

**FIGURE 3:**
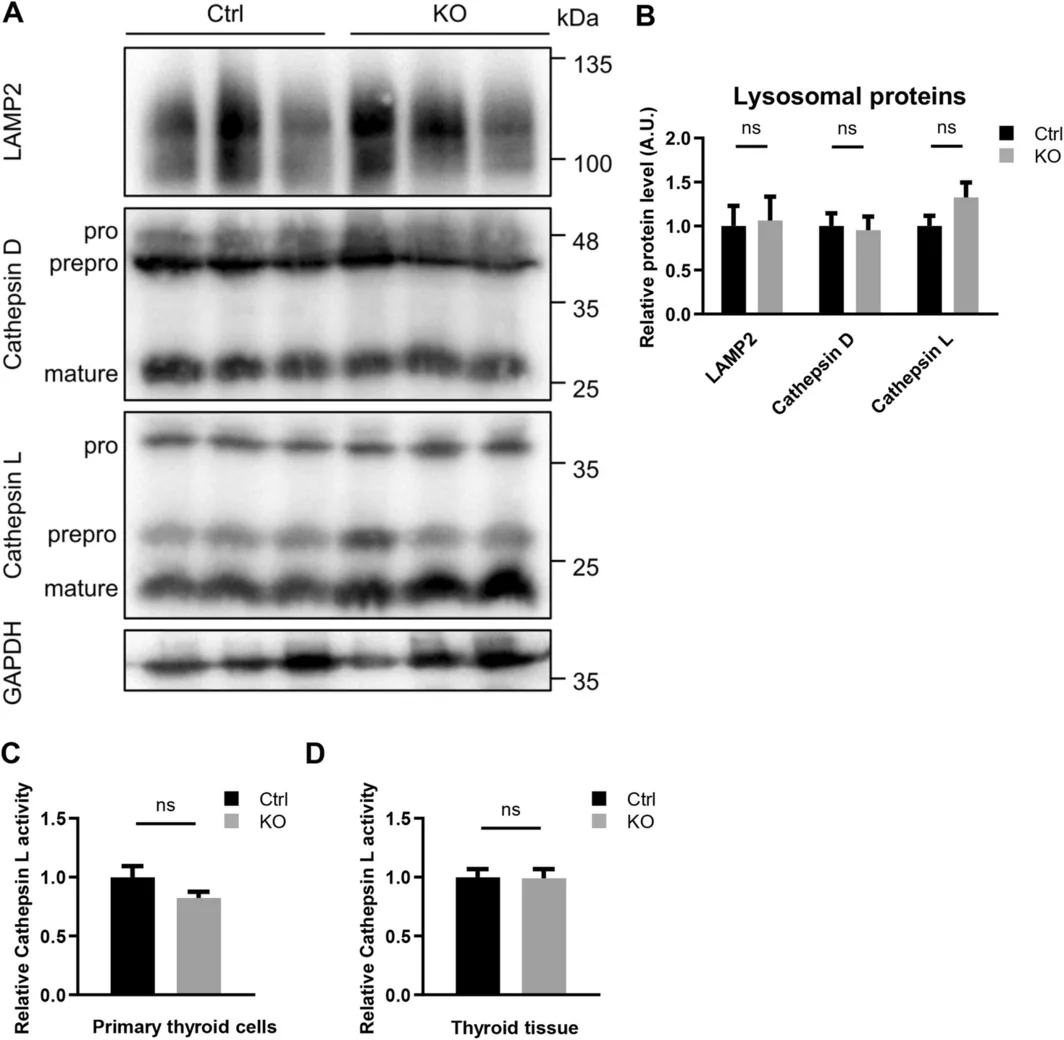
Analysis of lysosomal function in the *Mroh1*‐deficient thyroid. (A) Immunoblot analysis of lysosomal proteins. Representative immunoblots for LAMP2, cathepsin D, and cathepsin L in thyroid tissue lysates from Ctrl and KO mice at 3 months of age. GAPDH served as the loading control. Multiple bands corresponding to the pro‐, prepro‐, and mature forms of cathepsins D and L are indicated. (B) Quantification of lysosomal protein levels. Relative protein levels of the mature forms of LAMP2, cathepsin D, and cathepsin L normalized to GAPDH (*n* = 3 per group). (C) Intracellular cathepsin L activity. Live‐cell cathepsin L activity in primary thyroid cells, measured using the Magic Red fluorogenic substrate (*n* = 12). (D) Cathepsin L activity in tissue lysates. Enzymatic activity of cathepsin L quantified in whole‐thyroid tissue lysates from Ctrl and KO mice (*n* = 15). Data are presented as the mean ±SEM. Statistical significance was determined using Student's *t*‐test. ns, not significant. Ctrl, control; GAPDH, glyceraldehyde‐3‐phosphate dehydrogenase; KO, knockout; LAMP2, lysosome‐associated membrane protein 2.

Based on these findings, we hypothesized that the mild hypothyroidism observed in *Mroh1*‐KO mice might stem from impaired lysosomal biogenesis or enzyme activity, thereby disrupting Tg degradation. We first examined the expression levels of key lysosome‐associated proteins in the thyroid. Immunoblot analysis revealed that the protein levels of LAMP2 and the major lysosomal proteases, cathepsin L and cathepsin D, were not significantly different between Ctrl and KO mice (Figure 3A,B).

Because protein abundance does not necessarily reflect functional enzymatic activity, we next assessed lysosomal protease activity using two different approaches. First, a live‐cell assay using Magic Red, a fluorogenic substrate cleaved by cathepsin L, revealed no significant difference in intracellular cathepsin L activity between the primary thyroid cells derived from Ctrl and KO mice (Figure 3C). Furthermore, we quantified cathepsin L activity in whole‐thyroid tissue lysates. In agreement with the live‐cell assay, the enzymatic activity in the tissue lysates showed no significant difference between the genotypes (Figure 3D).

These results indicate that MROH1 deficiency does not affect lysosomal biogenesis or the proteolytic activity of lysosomal enzymes in mice. Although localized defects in intracellular localization or specific membrane trafficking events cannot be entirely ruled out, the fundamental capacity for lysosomal degradation is preserved in the *Mroh1*‐deficient thyroid.

## 4 Discussion

We demonstrate that global *Mroh1* deficiency results in reduced circulating thyroid hormone levels accompanied by late‐onset structural remodelling of the thyroid gland and secondary hypercholesterolemia. A key molecular feature of this phenotype is the transcriptional downregulation of thyroid lineage regulators (*Nkx2‐1* and *Foxe1*, with similar trends for *Pax8* and *Hhex*) together with reduced expression of thyroid functional genes, including *Tg* and *Mct8*. These changes are accompanied by architectural remodelling of the thyroid gland at 6 months of age, in which follicular structures are diminished. Collectively, these findings suggest that MROH1 is important for long‐term maintenance of thyroid follicular integrity and differentiated function in vivo.

A recent study by Li et al. established MROH1 as a regulator of lysosomal trafficking and integrity, showing that its depletion disrupts lysosomal fission and impairs cathepsin activity in *C. elegans* and COS‐7 cells [2]. Given the essential role of lysosomal proteases in Tg degradation and hormone liberation [15, 16], we first hypothesized that *Mroh1* deficiency would impair lysosomal function in the thyroid. Contrary to our expectation, lysosomal biogenesis and cathepsin L protease activity were largely preserved in the thyroid of *Mroh1*‐KO mice (Figure 3). This discrepancy may stem from differences in experimental systems (in vitro cell lines vs. in vivo tissue) or methodological sensitivity, as bulk tissue lysates and fluorogenic substrate assays may not capture localized alterations in lysosomal tubular dynamics. Furthermore, because we assessed overall lysosomal enzyme levels and activities rather than Tg cleavage products directly, we cannot completely rule out subtle defects in Tg processing or endo‐lysosomal trafficking. Direct evaluation of Tg degradation will be an important avenue for future research.

In this study, we suggest that the aetiology of the reduced circulating thyroid hormone levels in this mouse model is unlikely to be lysosomal dysfunction, but rather the transcriptional downregulation of thyroid‐specific genes mediated by reduced expression of transcription factors such as *Nkx2‐1* and *Foxe1* (Figure 2D).

Nevertheless, a critical distinction exists between our model and complete knockout models of MCT8 or Tg, which develop profound hypothyroidism accompanied by severe goitre (Table S4). In contrast, *Mroh1*‐KO mice exhibit a milder endocrine defect without thyroid enlargement, indicating that partial downregulation of these genes may lead to a distinct pathological presentation. Although the precise mechanism connecting MROH1 deficiency to these transcriptomic shifts remains to be clarified, adult‐stage ablation of *Foxe1* or *Nkx2‐1* is well known to suppress key thyrocyte differentiation markers, particularly *Tg* [17, 18]. The concurrent reduction in NKX2‐1 and FOXE1 is thus consistent with the intrinsic downregulation of thyroid functional genes. However, given that *Mroh1*‐KO mice carry a congenital gene deletion yet display a late‐onset phenotype, these thyroid‐intrinsic transcriptional alterations alone may be insufficient to explain the delayed pathology, and the potential contribution of extrathyroidal factors cannot be ruled out.

Although *Mroh1*‐KO mice in our study exhibited a mild hypothyroid phenotype with reduced circulating FT4 and FT3 levels, serum TSH levels showed no statistically significant difference compared with controls (Figure 1J). According to established guidelines on rodent thyroid economy [19], a reduction in circulating thyroid hormones typically triggers a compensatory elevation of serum TSH. The absence of a significant TSH response remains an unresolved feature and an important limitation of this study. The current data do not distinguish among altered central feedback, differences in hormone metabolism or transport.

Further functional assessment of the hypothalamic–pituitary system will be important in future studies to evaluate this central feedback response.

Although our tissue‐level assays did not reveal overt lysosomal enzyme dysfunction, these baseline measurements do not fully rule out the possibility of subtle, localized endo‐lysosomal trafficking defects due to limits in assay sensitivity. Because MROH1 is a conserved lysosomal fission factor, minor impairments in lysosomal membrane dynamics may gradually compromise cell function over time [2, 3]. As demonstrated in murine models of lysosomal storage diseases, such as infantile cystinosis (*Ctns*‐KO) and Fabry disease (*Gla*‐KO), lysosomal abnormalities often exhibit a prolonged lag phase, leading to age‐dependent, late‐onset tissue alterations [20, 21]. In *Ctns*‐KO mice, for example, compensated primary hypothyroidism and thyroid architectural remodelling manifest only after 9 months of age due to impaired lysosomal thyroglobulin processing and accumulated cellular stress [20]. A similar age‐dependent mechanism may operate in *Mroh1*‐KO thyrocytes, where minor endo‐lysosomal trafficking defects, undetected by standard bulk assays, gradually compromise long‐term thyroid gland homeostasis.

To clarify the metabolic features, we confirmed that body length (naso‐anal length) was unchanged in *Mroh1*‐KO mice at 24 weeks of age (Figure 1G), demonstrating that the body weight gain occurred independently of altered longitudinal skeletal growth. While hypercholesterolemia is a well‐established consequence of reduced thyroid hormone action, the mechanisms underlying body weight regulation in rodents are more complex. Unlike the frank weight gain typically observed in human hypothyroidism, mouse models with mild thyroid hormone deficiency do not consistently exhibit weight increases [22, 23], but rather have been reported to undergo subtle shifts in body composition. Therefore, the modest weight gain observed in *Mroh1*‐KO mice likely reflects systemic, extrathyroidal metabolic adaptations rather than a primary thyroidal effect.

In conclusion, we provide the first in vivo evidence demonstrating that MROH1 is an important factor for maintaining mammalian thyroid function and structural integrity. Future studies using tissue‐specific KO mouse models will be necessary to dissect the global versus thyroid‐specific contributions of MROH1.

## Author Contributions

**Nami Ohuchi:** investigation, formal analysis, methodology, software, data curation, writing – original draft. **Yoshinori Osaki:** conceptualization, project administration, supervision, writing – review and editing, validation, investigation, methodology. **Yoshimi Nakagawa:** writing – review and editing, supervision. **Takafumi Miyamoto:** writing – review and editing, visualization, funding acquisition. **Masaya Araki:** writing – review and editing. **Yuhei Mizunoe:** writing – review and editing. **Takaaki Matsuda:** writing – review and editing. **Yuki Murayama:** writing – review and editing. **Yoko Sugano:** writing – review and editing. **Hitoshi Iwasaki:** writing – review and editing. **Takashi Matsuzaka:** writing – review and editing, supervision. **Motohiro Sekiya:** writing – review and editing, supervision. **Hitoshi Shimano:** conceptualization, project administration, supervision, writing – review and editing.

## Funding

This research was supported by AMED under grant number JP256f0137008 to Takafumi Miyamoto.

## Conflicts of Interest

The authors declare no conflicts of interest.

## Supporting information


**Figure S1:** Serum biochemical parameters in Ctrl and KO mice. Serum levels of (A) AST, (B) ALT, (C) creatinine, (D) creatine kinase (CK), and (E) blood glucose in Ctrl and KO mice at 3 and 6 months of age. Data are presented as the mean ± SEM (*n* = 3–7 per group). Statistical significance was determined by Student's *t*‐test. ALT, alanine aminotransferase; AST, aspartate aminotransferase; CK, creatine kinase; Ctrl, control; KO, knockout; ns, not significant.

**Figure S2:** Histological analysis of various tissues in Ctrl and KO mice. Representative images of H&E‐stained sections from (A) the lung, (B) the brain (hippocampus), (C) the liver, and (D) the skeletal muscle (tibialis anterior) of Ctrl and KO mice at 6–9 months of age. No obvious histological abnormalities or structural changes were observed in any of these organs in KO mice compared to Ctrl mice. Scale bar = 100 μm. Ctrl, control; H&E, haematoxylin and eosin; KO, knockout; TA, tibialis anterior muscle.

**Table S1:** Primers used for mouse genotyping. Specific primer sequences for identifying the *Mroh1*‐floxed allele are listed. Genomic DNA was extracted from tail snips for the analysis. All sequences are shown in the 5′ to 3′ direction.
**Table S2:** Primers used for RT‐qPCR. Primers used for quantitative reverse transcription‐PCR (RT‐qPCR) of thyroid‐related genes, transcription factors, and the internal control are listed. All sequences are shown in the 5′ to 3′ direction.
**Table S3:** Antibodies used in this study.
**Table S4:** Comparison of endocrine and morphological features between *Mroh1*‐KO mice and previously reported thyroid‐related knockout mouse models.

## Data Availability

Data will be made available on request: This study generated *Mroh1* knockout mice. The mice will be made available on request.
