# Supplementary material for: The HEAT Repeat Protein MROH1 Deficiency Leads to Reduced Circulating Thyroid Hormone Levels in Mice
Source: Endocrinol Diabetes Metab. 2026 Sep 18;9(5):e70348. doi: 10.1002/edm2.70348 (PMC13587356; doi:10.1002/edm2.70348)
Supplement: Supplementary file 1 [file EDM2-9-e70348-s003.docx]

**Table S1. Primers used for mouse genotyping.**

Specific primer sequences for identifying the *Mroh1*-floxed allele are listed. Genomic DNA was extracted from tail snips for the analysis. All sequences are shown in the 5' to 3' direction.

| Target Gene(Alias) | Primer Name | Sequence (5' → 3') |
| --- | --- | --- |
| Mroh1 | Mroh1_282809_F  Mroh1_282809_R | TTGCTGACACCTATGCCCC  TTTAGAAGGGCCGAGACCAG |

**Table S2. Primers used for RT-qPCR**

Primers used for quantitative reverse transcription-PCR (RT-qPCR) of thyroid-related genes, transcription factors, and the internal control are listed. All sequences are shown in the 5' to 3' direction.

| Target Gene(Alias) | Primer Name | Sequence (5' → 3') |
| --- | --- | --- |
| Tg | Tg_Fw  Tg_Rv | TCCGGAGGAAAGTTGTGCTG  GCCGCTCACACTCAAAGAAC |
| Tpo | Tpo_Fw  Tpo_Rv | TGGATAGTAATTTGCAGGTGGACA  CCTCTATTCGCACAGGAGGAC |
| Tshr | Tshr_Fw  Tshr_Rv | ATGACACGGAAGTGGCTCTG  ACTCTGCACTTCTCTCTCCTTC |
| Slc5a5 (Nis) | Slc5a5 (Nis)_Fw  Slc5a5 (Nis)_Rv | GGGATGCACCAATGCCTCTG  GTAGCTGATGAGAGCACCACA |
| Slc16a2 (Mct8) | Slc16a2 (Mct8)_Fw  Slc16a2 (Mct8)_Rv | CGGCTGGATAGTGGTGTTTG  CAGAGTTATGGATGCCGAAGATG |
| Slc26a4 (Pendrin) | Slc26a4 (Pendrin)_Fw  Slc26a4 (Pendrin)_Rv | GCTGGCCTCATCTCAGCTG  GCAAGGGTTCCAGAAGCCT |
| Slc26a7 | Slc26a7_Fw  Slc26a7_Rv | CCCCACCGAGAAGACATTAAGC  TGAACTGCCAACATTATCCCAG |
| Pax8 | Pax8_Fw  Pax8_Rv | CAGCCTGCTGAGTTCTCCAT  CTGTCTCAGGCCAAGTCCTC |
| Nkx2-1 | Nkx2-1 (Ttf-1)_Fw  Nkx2-1 (Ttf-1)_Rv | CGCCTTACCAGGACACCAT  CCCATGCCACTCATATTCAT |
| Hhex | Hhex_Fw  Hhex_Rv | AAGTGAGGTTCTCCAACGACC  CATTTAGCTCGGCGATTCTGAA |
| Foxe1 | Foxe1 (Ttf-2)_Fw  Foxe1 (Ttf-2)_Rv | GGCGGCATCTACAAGTTCAT  GGATCTTGAGGAAGCAGTCG |
| Mroh1 | Mroh1_Fw  Mroh1_Rv | TCCTTTTCTGCCGTCCATCC  CCCCAAAGGTGTCCTTCCTC |
| Mroh2a | Mroh2a_Fw  Mroh2a_Rv | CTCTGTCCCGAAACCACTTCA  GTGACAATGACAAACTCGTCAGT |
| Gapdh | Gapdh_Fw  Gapdh_Rv | GTCGTGGATCTGACGTGCC  ATGCCTGCTTCACCACCTTC |

**Supplementary Table S3. Antibodies used in this study**

| Target (Antigen) | Manufacturer | Catalog No | RRID |
| --- | --- | --- | --- |
| LAMP2 | DSHB | ABL-93 | AB_2134767 |
| Cathepsin D | R&D Systems | AF1029 | AB_2087094 |
| Cathepsin L | R&D Systems | AF1515 | AB_2087690 |
| GAPDH | Santa Cruz | sc-32233 | AB_627679 |
| HRP-conjugated goat anti-rabbit IgG | Cell Signaling Technology | 7074S | AB_2099233 |
| HRP-conjugated horse anti-mouse IgG | Cell Signaling Technology | 7076S | AB_330924 |
| HRP-conjugated donkey anti-goat IgG | R&D Systems | HAF109 | AB_357236 |

Antibodies used for western blotting are listed with their manufacturers and catalog numbers. Abbreviations: HRP, horseradish peroxidase; IgG, immunoglobulin G.

**Supplementary Table S4. Comparison of endocrine and morphological features between *Mroh1*-KO mice and previously reported thyroid-related knockout mouse models.**

|  | *Mct8-*KO [1] | *Tg-*KO [2, 3] | *Tg* ^+/−^ [2] | *Mroh1*-KO  (Present study) |
| --- | --- | --- | --- | --- |
| Serum FT4 or T4 | Low | Very Low (Undetectable) | Normal | **Slightly Low** |
| Serum FT3 or T3 | High* | Low | No data | **Slightly Low** |
| Serum TSH | Mildly High | Extremely High (>5,000 mU/L) | Normal | **Unchanged** |
| Body Weight | Unchanged | Severe Growth Impairment (Dwarfism) | Normal | **Slightly Increased** |
| Thyroid Gland Morphology | Enlarged  (1.6-fold) | Massive Goiter  (5-fold) | Normal | **Normal Overall Size (Shift toward smaller follicles)** |

Values in bold represent findings from the present study. *Tg*^+/-^ indicates heterozygous *Tg*-knockout mice. Abbreviations: FT4, free thyroxine; FT3, free triiodothyronine; TSH, thyroid-stimulating hormone; KO, knockout;

**References**

1.	Di Cosmo C, Liao XH, Dumitrescu AM, Philp NJ, Weiss RE, Refetoff S. Mice deficient in MCT8 reveal a mechanism regulating thyroid hormone secretion. J Clin Invest. 2010;120(9):3377-88.

2.	Young C, Zhang X, Wang X, Kellogg AP, Pena K, Cumming AZ, et al. Stimulated thyroid hormone synthesis machinery drives thyrocyte cell death independent of ER stress. J Clin Invest. 2025;135(24).

3.	Young C, Zhang X, Kellogg AP, Fonseca TL, Pena K, Beyzin A, et al. Thyroid Hormone Synthesis Without Thyroglobulin. Thyroid : official journal of the American Thyroid Association. 2026;36(4):441-50.
